# Adverse drug reactions in neonates: a brief analysis of the FDA adverse event reporting system

**DOI:** 10.3389/fphar.2024.1395982

**Published:** 2024-06-06

**Authors:** Pernille Kähler Byskov, Christoffer Storm Baden, Jon Trærup Andersen, Espen Jimenez-Solem, Ramus Huan Olsen, Christina Gade, Ulrik Lausten-Thomsen

**Affiliations:** ^1^ Department of Clinical Pharmacology, Copenhagen University Hospital–Bispebjerg and Frederiksberg, Copenhagen, Denmark; ^2^ Department of Clinical Medicine, University of Copenhagen, Copenhagen, Denmark; ^3^ Department of Neonatology, Copenhagen University Hospital–Rigshospitalet, Copenhagen, Denmark

**Keywords:** pharmacology, adverse drug events, neonatal pharmacology, public health, pharmacovigilance

## Abstract

**Introduction:**

Drug trials in neonates are scarce, and the neonates may consequently be at risk of adverse drug reactions (ADRs). Spontaneous ADR reporting is an important tool for expanding the knowledge on drug safety in neonates. This study explores the quality of current neonatal ADR reports and the ADR reports of the most common drugs used in neonatal departments.

**Methods:**

An observational cross-sectional study focused on neonates was conducted using data on spontaneous reports extracted from the U.S. Food and Drug Administration Adverse Events Reporting System (FAERS) from the third quarter of 2014 up to December 2022. Only the primary suspect drugs given to neonates or subjects aged <30 days were included in the analysis.

**Results:**

Spontaneous reports from 13 million patients of all ages, totaling 50 million ADRs, were evaluated. Information regarding the age was missing in 40% of the reports, and data on 43,737 neonates with 948 different suspected drugs were identified and included in the analysis. We report the frequency of spontaneous ADR reports in the FAERS database for the ten most frequently administered drugs in neonatal intensive care units in the USA.

**Conclusion:**

Overall, neonatal ADRs are still underreported. The FAERS database in its current form discriminates insufficiently between prenatal and postnatal drug exposures. Hence, improved neonatal pharmacovigilance systems are urgently needed.

## Introduction

Drug trials in neonates are scarce; as a consequence, up to 90% of the treatments in neonates are off-label, raising concerns about the risks of adverse drug reactions (ADRs) (Stark et al., 2021; Gade et al., 2023). Given the limited clinical testing in such instances, spontaneous ADR reporting is an important tool for expanding knowledge on and increasing drug safety in neonates; however, it is well known that the applicability of the currently available data is undermined by underreporting (Khalili et al., 2020). The pharmacovigilance value and ADR signal-generating capacities of the registers of collected data are no better than the quality of the data in these registers. Often, data on the age and weight are missing, and data on prenatal and postnatal exposures are often combined, limiting the usefulness of the registers from the perspective of neonatal pharmacology. Accordingly, this study aims to explore the quality of the current neonatal ADR reports in the U.S. Food and Drug Administration Adverse Events Reporting System (FAERS) database and to explore the most commonly reported drugs causing ADRs in neonates.

## Materials and methods

An observational cross-sectional study focused on neonates was conducted using the data extracted from the FAERS. The study included spontaneous reports from the inception of FAERS in the third quarter of 2014 to 31 December 2022. Only the primary suspect drugs given to patients coded as neonates or subjects aged <30 days were included in the analysis, categorized based on active substances, and further stratified by intrauterine or extrauterine exposure. Intrauterine exposure was defined based on ADR reports from i) transplacental administration route, ii) indications of maternal/fetal exposure, and iii) listings having congenital anomalies as the outcomes. To distinguish between neonatal ADRs through maternal/fetal and direct exposure, the most frequently administered drugs in neonatal intensive care units in the US were investigated specifically, as per the estimation by Stark et al. (2021).

## Results

The study period spanning 8.5 years encompassed spontaneous reports from 13 million patients of all ages, totaling 50 million ADRs in the FAERS. Information regarding the age was missing in 40% of these reports, and data on 43,737 neonates with 948 different suspected drugs were finally identified and included in the analysis. Table 1 presents the drugs most frequently reported alongside the suspected patient outcomes. It is worth noting that the ADRs are often linked with intrauterine rather than extrauterine exposure of the neonate, consistent with birth defects comprising 30% of all reported ADRs; indeed, most of the drugs reported in Table 1 as neonatal are most likely maternal exposure. Table 2 presents the frequency of spontaneous ADR reports in the FAERS database for the ten most frequently administered drugs in neonatal intensive care units in the US, as reported by Stark et al. (2021). Detailed demographic information (e.g., gestational age, sex, postmenstrual age of the mother, and race/ethnicity) on the included infants was not available in the FAERS, precluding analyses on ADRs in relation to specific demographic factors.

**TABLE 1:** Top 10 drugs reported to the FAERS database for the neonatal population during 2014–2022^d^.

Primary suspected drug		Number of adverse drug reactions (ADRs) reported	Patient outcome of ADR (%)^a^ Ranked by outcome resulting in each ADR to be included once Death > Life threatening > Hospitalization > Disability > Birth defect > Other serious medical event > Not reported
	All reported drugs	43737	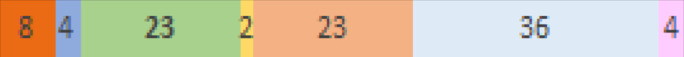
1	Hydroxyprogesterone^b^ ^,^ ^c^	3,068	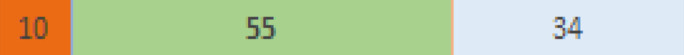
2	Oxycodone^b^	2,480	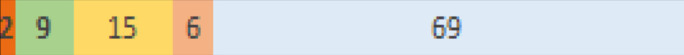
3	Levetiracetam	1,302	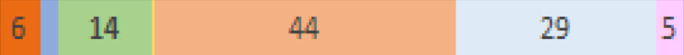
4	Lamotrigine^b^	986	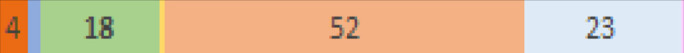
5	Palivizumab	937	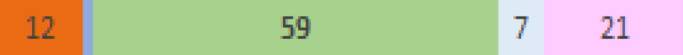
6	Ondansetron^b^	791	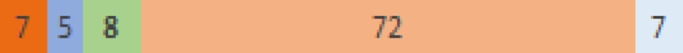
7	Buprenorphine^b^	738	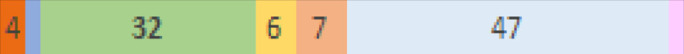
8	Sertraline^b^	732	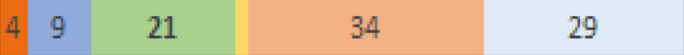
9	Venlafaxine^b^	699	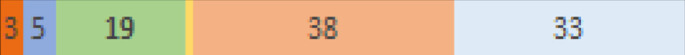
10	Quetiapine^b^	673	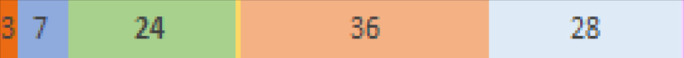

^a^ The outcome “Requiring intervention” had no reports.

^b^ Assumed intrauterine exposure, with the mother as the desired recipient.

^c^ Assumed intrauterine exposure, with the fetus as the desired recipient.

*Some of these drugs are likely to represent maternal pharmacotherapy; see text for details.

**TABLE 2:** Top 10 most frequent drugs used in neonatal intensive care units in the US and the corresponding drug reports in the FAERS database.

Drugs most frequently used in the neonatal intensive care unit ranked by exposure. Frequency of hospitalized neonates exposed to the drug (%) as per Stark et al.^a^		Spontaneously reported adverse drug reactions (ADRs) to the FAERS		
		Intrauterine exposure	Extrauterine exposure	Patient outcome of ADR (%)^b^ Ranked by outcome resulting in each adverse drug event to be included once Death > Life threatening > Hospitalization > Disability > Birth defect > Other serious medical event > Not reported
1	Ampicillin (58.2)	41	157	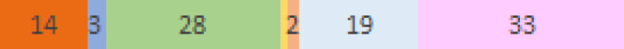
2	Gentamicin (57.9)	2	148	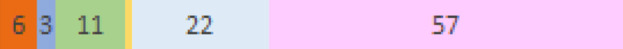
3	Caffeine citrate (15.3)	0	13	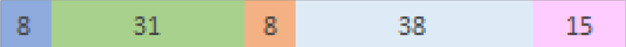
4	Poractant alfa (8.9)	0	308	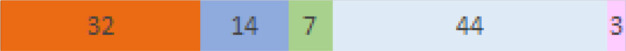
5	Morphine (6.8)	188	138	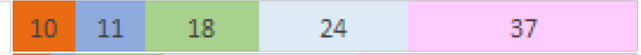
6	Vancomycin (6.1)	19	285	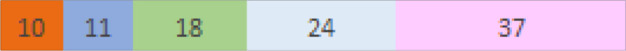
7	Furosemide (5.8)	70	150	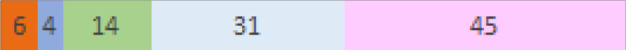
8	Fentanyl (5.6)	174	104	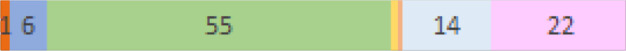
9	Midazolam (4.7)	1	25	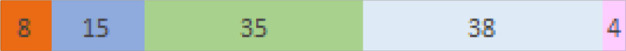
10	Acetaminophen (4.2)	226	150	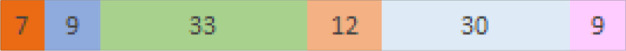

^a^ Reference: Stark et al., Medication Use in the Neonatal Intensive Care Unit and Changes from 2010 to 2018. J Pediatr 2021:S0022-3,476 (21)00860-X.

^b^ The outcome “Requiring intervention” had no reports.

## Discussion

Compared to all other age groups, neonates have the highest proportion of serious ADRs reported for both the overall and all subcategories of seriousness, with the exemption of death and disability in infants below 2 years of age, as well as underreporting (Phan et al., 2023). This observation is cemented in the present study. According to data from the U.S. Centers for Disease Control (CDC) Wide-Ranging Online Data for Epidemiologic Research (WONDER) database, approximately 9.3% of newborns in the US are admitted to a neonatal department, which is equivalent to approximately 3 million neonates over the study period. For perspective, as few as 1.5% of the hospitalized neonates were estimated to have ADRs reported during the study period, upon comparing the data in the present study (*n* = 43,737) with data from the WONDER database. In this estimate, we assume that all reported ADRs were observed during hospitalization, which is reasonable since drug administration in neonates primarily occurs within a hospital setting; however, this estimate is not adjusted for the number of hospitalized neonates that only received non-pharmacological treatment. Furthermore, as listed in Table 1, the majority of the reported ADRs in the FAERS database are attributed to drugs administered to the expecting mothers, implying that the reporting rate for ADRs associated exclusively with neonatal treatments is extremely low.

Therefore, the results illustrate an overall significant underreporting of adverse events in neonates, which supports the findings of previous studies (Hawcutt et al., 2016). This underreporting can be exemplified in isolation by observing data for a frequently administered drug with a well-known narrow therapeutic index, such as gentamicin (Kent et al., 2014). Gentamicin is used to treat severe infections and is administered to approximately half of all newborns admitted to a neonatal department in the US (Stark et al., 2021). Gentamicin is well-known to be associated with numerous serious ADRs, including nephrotoxicity and ototoxicity. A meta-analysis based on six studies in the neonatal population estimated that ototoxicity occurs in 3% (95% confidence interval: 0%–7%) of the gentamicin-treated neonates (Musiime et al., 2015) and that nephrotoxicity, although not observed in all studies, has been reported in up to 27% of the cases in some studies (McWilliam et al., 2017). However, in the present study of reported neonatal ADRs, we identify a mere 148 spontaneous reports (Table 2) from the estimated 1.5 million neonates exposed to gentamicin (0.01%), suggesting a gross underreporting of gentamicin-associated ADRs in general.

Underreporting of ADRs is primarily attributable to in the inherent difficulties in identifying ADRs in newborns because of their immature organ systems, in addition to a general lack of attention to ADRs and the shortage of allocated resources. In the above example, gentamicin-associated toxicity may be difficult to identify in neonates as hearing loss after a severe bacterial infection is often multifactorial in origin. Furthermore, the difficulty may be a result of the longer follow-up time required for quantitative vestibular function testing and complexity of the assessment (Musiime et al., 2015). Still, this example emphasizes the importance of a low threshold for reporting ADRs and based on suspicions alone, while the final assessment of causality should be conducted by the health authorities. Although it is important to stress that a given ADR report does not necessarily reflect causality, such spontaneous reports are paramount for generating ADR signals and pharmacosurveillance. Failure to recognize and report ADRs can significantly increase the risk of both subtherapeutic and supratherapeutic treatments in neonates as well as toxicity that may lead to disabilities or death in the worst case.

Although there have been improvements in the limit for early human viability in recent decades and consequently an increase in complex pharmacological treatments in neonatal intensive care units, the ADR reporting approach appears to be just as limited as it was 20 years ago (Le et al., 2006). Systematic collection of safety data and protocolized treatments must therefore be introduced as standards in every neonatal intensive care unit as soon as possible. Otherwise, it is difficult to ensure drug safety in this vulnerable population that still lives in a pharmacologically unregulated “no man’s land.”

The FAERS is one of the largest spontaneous ADR reporting databases in the world, which makes it the obvious choice for pharmacovigilance research. However, the FEARS has several shortcomings in relation to practices reporting ADRs in neonates; for example, many reports do not include the gestational age or even the age at the time of exposure. Furthermore, it is often difficult to determine whether drug exposure occurred during the intrauterine or neonatal period, as demonstrated in the present study. Intended prenatal exposure through maternal pharmacotherapy, such as the maternal administration of betamethasone to increase fetal/neonatal lung maturation, and non-intended fetal exposure, such as maternal psychopharmacotherapy, are difficult to separate in the current form. Although prenatal exposure is subject to some degree of pharmacovigilance, such as through pregnancy registers, there is an ongoing need for improving neonatal pharmacovigilance, particularly in light of the widespread neonatal off-label pharmacotherapy, warranting evaluation of the current databases that track neonatal pharmacotherapy. It is important to stress that these examples obviously introduce challenges in the data analyses and may ultimately lead to inaccurate data reporting.

The findings of this study demonstrate that the FEARS database in its current form has insufficient neonatal (and possibly even pediatric) pharmacovigilance as the data on subject age was missing in about 40% of the reports. Mandatory age reporting on all ADR reports and/or mandatory distinction of maternal/fetal exposure in any report on children under the age of say 1 year would be an instant and cost-effective quality improvement of the pediatric/neonatal applicability of the current reporting system. On a practical note, this can be achieved through the use of well-designed, intuitive, and user-friendly online solutions that offer a relatively inexpensive method of improving data as these online-based solutions could incorporate reminders to remember stating the subject age when reporting an ADR. Moreover, additional pop-up questions relevant to pediatrics (such as weight, maternal/pediatric exposure, and gestational age at birth) could be presented as a guide to the reporter based on age-appropriateness.

From the perspective of the reporter, numerous improvements have been proposed (Allegaert and van den Anker, 2015; Phan et al., 2023) regarding awareness of ADRs, including clinical “trigger tools” aimed at identifying both clinical adverse events as well as ADRs; however, these are far from being operationally implemented in neonatal intensive care units and need to be refined to better support the health personnel in identifying ADRs (Sharek et al., 2006). We are of the opinion that it is crucial to prioritize training (such as using the neonatal adverse event severity scale) while bolstering active surveillance efforts (such as sentinel sites/departments or focusing on drugs of special interest) to improve ADR detection and reporting effectively (Salaets et al., 2019).

State health departments can also provide guidelines and resources to support these efforts. At the federal scale, agencies like the FDA can mandate the inclusion of neonatal ADR data in post-marketing surveillance and encourage research to fill the gaps in knowledge. Without gathering comprehensive knowledge on ADRs in often neglected populations, the benefit–risk assessments of pharmacological treatments become extremely difficult. The same applies to our understanding of drugs used off-label, which can be improved through careful reporting of ADRs. This area should be prioritized at the clinical and decision-making levels.

## Data Availability

Publicly available datasets were analyzed in this study. These data can be found at: https://fis.fda.gov/sense/app/95239e26-e0be-42d9-a960-9a5f7f1c25ee/sheet/7a47a261-d58b-4203-a8aa-6d3021737452/state/analysis.

## References

[B1] Allegaert K. van den Anker J. N. (2015). Adverse drug reactions in neonates and infants: a population-tailored approach is needed. Br. J. Clin. Pharmacol. 80, 788–795. 10.1111/bcp.12430 24862557 PMC4594721

[B2] Gade C. Trolle S. Mørk M.-L. Lewis A. Andersen P. F. Jacobsen T. (2023). Massive presence of off-label medicines in Danish neonatal departments: a nationwide survey using national hospital purchase data. Pharmacol. Res. Perspect. 11, e01037. 10.1002/prp2.1037 36545691 PMC9772727

[B3] Hawcutt D. B. Russell N.-J. Maqsood H. Kouranloo K. Gomberg S. Waitt C. (2016). Spontaneous adverse drug reaction reports for neonates and infants in the UK 2001-2010: content and utility analysis. Br. J. Clin. Pharmacol. 82, 1601–1612. 10.1111/bcp.13067 27597136 PMC5099553

[B4] Kent A. Turner M. A. Sharland M. Heath P. T. (2014). Aminoglycoside toxicity in neonates: something to worry about? Expert Rev. Anti Infect. Ther. 12, 319–331. 10.1586/14787210.2014.878648 24455994

[B5] Khalili M. Mesgarpour B. Sharifi H. Daneshvar Dehnavi S. Haghdoost A. A. (2020). Interventions to improve adverse drug reaction reporting: a scoping review. Pharmacoepidemiol Drug Saf. 29, 965–992. 10.1002/pds.4966 32431069

[B6] Le J. Nguyen T. Law A. V. Hodding J. (2006). Adverse drug reactions among children over a 10-year period. Pediatrics 118, 555–562. 10.1542/peds.2005-2429 16882807

[B7] McWilliam S. J. Antoine D. J. Smyth R. L. Pirmohamed M. (2017). Aminoglycoside-induced nephrotoxicity in children. Pediatr. Nephrol. 32, 2015–2025. 10.1007/s00467-016-3533-z 27848094 PMC5624973

[B8] Musiime G. M. Seale A. C. Moxon S. G. Lawn J. E. (2015). Risk of gentamicin toxicity in neonates treated for possible severe bacterial infection in low- and middle-income countries: systematic Review. Trop. Med. Int. Health 20, 1593–1606. 10.1111/tmi.12608 26426298

[B9] Phan M. Cheng C. Dang V. Wu E. Muñoz M. A. (2023). Characterization of pediatric reports in the US food and drug administration adverse event reporting system from 2010-2020: a cross-sectional study. Ther. Innov. Regul. Sci. 57, 1062–1073. 10.1007/s43441-023-00542-0 37351842 PMC10527885

[B10] Salaets T. Turner M. A. Short M. Ward R. M. Hokuto I. Ariagno R. L. (2019). Development of a neonatal adverse event severity scale through a Delphi consensus approach. Arch. Dis. Child. 104, 1167–1173. 10.1136/archdischild-2019-317399 31537552 PMC6943241

[B11] Sharek P. J. Horbar J. D. Mason W. Bisarya H. Thurm C. W. Suresh G. (2006). Adverse events in the neonatal intensive care unit: development, testing, and findings of an NICU-focused trigger tool to identify harm in North American NICUs. Pediatrics 118, 1332–1340. 10.1542/peds.2006-0565 17015521

[B12] Stark A. Smith P. B. Hornik C. P. Zimmerman K. O. Hornik C. D. Pradeep S. (2021). Medication use in the neonatal intensive care unit and Changes from 2010 to 2018. J. Pediatr. S0022-3476 (21), 66–71.e4. 10.1016/j.jpeds.2021.08.075 PMC9394450 34481808

